# Association of real life postural transitions kinematics with fatigue in neurodegenerative and immune diseases

**DOI:** 10.1038/s41746-024-01386-0

**Published:** 2025-01-06

**Authors:** Robbin Romijnders, Arash Atrsaei, Rana Zia Ur Rehman, Lea Strehlow, Jèrôme Massoud, Chloe Hinchliffe, Victoria Macrae, Kirsten Emmert, Ralf Reilmann, C. Janneke van der Woude, Geert Van Gassen, Frédéric Baribaud, Teemu Ahmaniemi, Meenakshi Chatterjee, Bruno Kusznir Vitturi, Clémence Pinaud, Jérôme Kalifa, Stefan Avey, Wan-Fai Ng, Clint Hansen, Nikolay V. Manyakov, Walter Maetzler

**Affiliations:** 1 Department of Neurology, University Hospital Schleswig-Holstein Campus Kiel, Kiel University, Kiel, Germany; 2 Mindmaze SA, Digital Motion Analytics Team, Lausanne, Switzerland; 3 Janssen, Johnson & Johnson, Newcastle Upon Tyne, UK; 4 Translational and Clinical Research Institute, Faculty of Medical Sciences, Newcastle University, Newcastle Upon Tyne, UK; 5 George-Huntington-Institute, Muenster, Germany; 6 Erasmus MC, Rotterdam, The Netherlands; 7 Medical Affairs, Takeda Belgium NV, Zaventem, Belgium; 8 Translational Development, Bristol Meyers Squibb, Spring House, PA USA; 9 VTT Technical Research Centre of Finland Ltd, Tampere, Finland; 10 Janssen Research & Development, Spring House, PA USA; 11 Let It Care, Paris, France; 12 HRB Clinical Research Facility Cork, University College Cork, Cork, Ireland; 13 Janssen Research & Development, Beerse, Belgium

**Keywords:** Diagnostic markers, Health care, Parkinson's disease

## Abstract

Fatigue is prevalent in immune-mediated inflammatory and neurodegenerative diseases, yet its assessment relies largely on patient-reported outcomes, which capture perception but not fluctuations over time. Wearable sensors, like inertial measurement units (IMUs), offer a way to monitor daily activities and evaluate functional capacity. This study investigates the relationship between sit-to-stand and stand-to-sit transitions and self-reported physical and mental fatigue in participants with Parkinson’s, Huntington’s, rheumatoid arthritis, systemic lupus erythematosus, primary Sjögren’s syndrome and inflammatory bowel disease. Over 4 weeks, participants wore an IMU and reported fatigue levels four times daily. Using mixed-effects models, associations were identified between fatigue and specific kinematic features, such as 5th and 95th percentiles of sit-to-stand performance, suggesting that fatigue alters the control and effort of movement. These kinematic features show promise as indicators for fatigue in these patient populations.

## Introduction

Fatigue is a subjective sensation of weakness, lack of energy or tiredness^1^. It is considered a multi-dimensional phenomenon in which the biophysiological, cognitive, motivational and emotional state of the body is affected, thereby significantly impairing an individual’s ability to function in their daily life^2,3^. Physical and mental aspects are most widely recognised, with physical fatigue being associated with a subjective lack of physical energy that interferes with usual and desired activities^3,4^, whereas mental fatigue is associated with a reduced efficiency in cognitive performance^3,5–7^. In neurodegenerative (NDD) and immune-mediated inflammatory diseases (IMID), fatigue is particularly common and has been rated among the symptoms having the most impact on health-related quality of life (HrQoL)^8–17^. In fact, the prevalence of fatigue in this group of diseases is high, ranging from one third to more than half of patients affected^18–20^. When talking about fatigue, it is important to distinguish between fatigue and fatigability. Fatigue refers to the subjective feeling, whereas fatigability is defined as ‘the magnitude or rate of change in a performance criterion relative to a reference value over a given time of task performance or measure of mechanical output’^1^.

The pathophysiology of fatigue is complex and not fully understood. However, evidence points to peripheral and systemic inflammation as key factors in both NDD and autoimmune diseases^21^. Current clinical assessment of fatigue relies heavily on patient-reported outcomes (PROs), that capture mainly how people perceive their fatigue^22^. PROs are subjective, prone to recall bias and poorly capture variability over time^2,23^. Objective assessment of fatigue using wearable sensors, such as inertial measurement units (IMUs), could help collect more and complementary information about fatigue than what we can get from PROs as sensor-derived parameters overcome the aforementioned issues. Wearable sensors allow continuous monitoring in the real world and are therefore able to capture symptom fluctuations, and such digital health measures are objective and are considered very sensitive to changes over time^24–27^. However, some degree of association is expected between parameters obtained from these measurements, as they measure the same concept of interest and thus offer us a ‘window of validation opportunity’ for novel assessment strategies. In that sense, novel digital measures will augment established PROs, rather than replacing them and ultimately paint a more complete picture of the patient’s burden^28^. In addition, sensor-derived parameters from continuous monitoring capture capacity and performance aspects of health^22,29–31^ thereby painting a more complete picture of the impact of fatigue on daily function. Furthermore, both the US Food and Drug Administration and the European Medicines Agency (EMA) encourage the inclusion of digital health measures from unsupervised, i.e. real-world, patient monitoring as surrogate or intermediate clinical endpoints in clinical trials^32–34^.

One of the mechanically most demanding activities of daily life, and a necessity of daily function, is rising from sitting to standing^35,36^. Furthermore, the ability to rise from sitting to standing and vice versa, is a prerequisite for functional independence^37^. These so-called postural transitions (PTs) are performed multiple times a day, e.g. on average people with Parkinson’s disease (PD) perform >3 sit-to-stand transition per hour^38^, and it was shown that measures of PT are indicators of overall functioning and balance in older adults^39,40^ and are associated with disability and morbidity^41,42^. Furthermore, kinematic features derived from these transitions discriminated older fallers from non-fallers, for example using sit-to-stand vertical velocity, power and acceleration^43^ or vertical velocity, acceleration, power and jerk^44^. Moreover, falls are associated with fatigue in both, older adults^45^ and people with PD^46,47^. Therefore, we hypothesised that kinematic features (e.g. duration, peak velocity, peak acceleration) of sit-to-stand and stand-to-sit transitions are associated with fatigue, and thus the aim of this study was to quantify the association between kinematic features of sit-to-stand and stand-to-sit transitions and self-reported physical and mental fatigue. It is expected that physical fatigue, which is associated with a subjective lack of physical energy that interferes with usual and desired activities, should affect parameters of muscle activity, for example for movements against gravity. Mental fatigue is expected to be reflected rather by movements that require intersegmental coordination, such as when sitting down.

## Results

### Study participants

For a total of 115 participants, kinematic features of sit-to-stand and stand-to-sit transitions were detected from the lower-back IMU. From those participants, there were 101 participants that also had filled out PROs, and finally there were 86 participants that had both PROs and IMU-derived kinematic features in any 2-h aggregation window (Table 1).

**Table 1:** Demographics data of the participants that filled out PROs and had postural transitions registered from the lower-back IMU within the 2-h aggregation window

			Age	Height	Weight
Group	Gender	*N*	years	m	kg
HD	female	5	41 (8)	1.68 (0.08)	83 (26)
	male	3	48 (15)	1.79 (0.07)	77 (10)
IBD	female	7	38 (13)	1.67 (0.02)	65 (5)
	male	6	36 (9)	1.85 (0.09)	81 (9)
PD	female	5	59 (14)	1.65 (0.08)	66 (14)
	male	4	59 (11)	1.82 (0.07)	76 (9)
PSS	female	9	62 (15)	1.60 (0.07)	66 (8)
	male	2	68 (6)	1.82 (0.03)	82 (16)
RA	female	10	59 (11)	1.62 (0.13)	77 (14)
	male	2	65 (1)	1.80 (0.05)	102 (16)
SLE	female	9	51 (15)	1.64 (0.07)	69 (13)
HV	female	12	48 (15)	1.68 (0.07)	75 (16)
	male	11	48 (14)	1.82 (0.07)	87 (14)

Age, height and weight are presented as mean (standard deviation).

*HD* Huntington’s disease, *HV* healthy volunteers, *IBD* inflammatory bowel disease, *PD* Parkinson’s disease, *PSS* primary Sjörgen’s syndrome, *RA* rheumatoid arthritis, *SLE* systemic lupus erythematosus.

### Patient-reported outcomes

In total, there were 1904 2-h windows centred around the diary responses, in which also postural transitions were detected. From these windows, 139 were excluded because there were fewer than 3 transitions. This resulted in 1765 windows that were used for analysis.

**Fig. 1:**
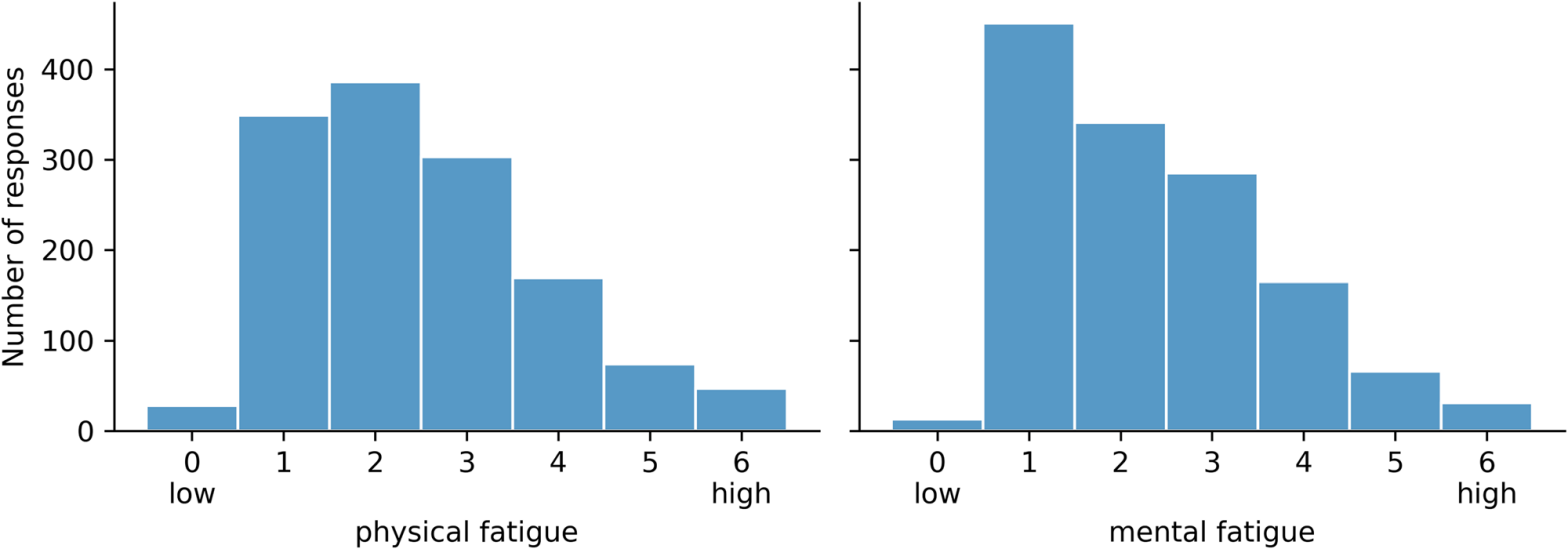
Histogram plots for the administered PROs. Distribution of the self-reported levels of self-perceived physical (left) and mental (right) fatigue. Note that the visualisation of the number of responses over the day showed similar distributions for both mental and physical fatigue (data not shown).

Exploratory analysis of the PROs revealed a right-skewed distribution of both physical and mental fatigue (Fig. 1). The distributions showed that only a small fraction of responses corresponded to the lowest fatigue (28 out of 1278 responses (2%) for physical fatigue, and 13 out of 1274 responses (1%) for mental fatigue) suggesting that most participants experienced at least some level of fatigue. The highest values of fatigue (scores 5 and 6) were reported in 111 out of 1278 responses (9%) for physical fatigue and in 92 out of 1274 responses (7%) for mental fatigue, suggesting that participants did also experience extreme values of fatigue.

### Associations between kinematic features and fatigue

The validated postural transition detection algorithm^48,49^ detected sit-to-stand and stand-to-sit transitions. The association between each kinematic feature and the self-reported fatigue was modelled with a mixed effects model with a random intercept and a fixed slope. Of particular interest was the estimate for the slope, since a statistically significant deviation from zero hints at an association between the kinematic feature and fatigue, which was identified by a *p*‐value below 0.05. For the slope estimates that showed statistical significance, an additional model was run with a random intercept and random slope.

For the sit-to-stand transitions, the relationship between the 95th percentile peak trunk angular velocity and physical fatigue, and the association between the 95th percentile trunk angular range and mental fatigue showed a slope that was statistically different from zero.

For stand-to-sit transitions, the 95th percentile PT duration and physical fatigue revealed a slope estimate that was significantly different from zero, and the 5th percentiles of peak trunk angular velocity, peak jerk and angular and physical fatigue.

**Fig. 2:**
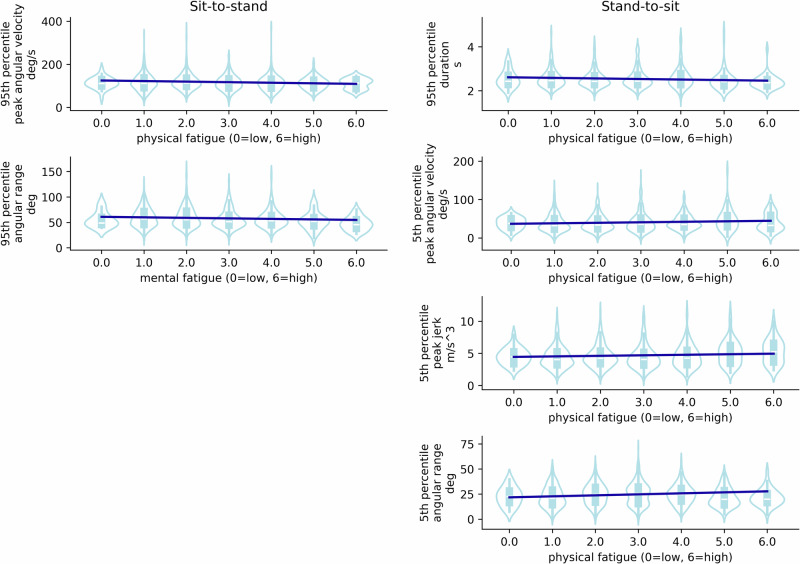
Scatter plots and linear fits for the kinematic features and physical and mental fatigue. Scatter plots and linear fits for the kinematic features that showed a significant estimate for the slope. The linear fit (blue line) represents the group fit. For the sit-to-stand transitions (left) the kinematic features were the 95th percentiles, whereas for the stand-to-sit transitions (right) they were the 5th percentiles, except for the duration (top right).

Scatter plots and a linear fit of the 95th percentile of the peak trunk angular velocity with physical fatigue, and the 95th percentile trunk angular range and mental fatigue for the sit-to-stand transitions showed a negative relationship (Table 2, Fig. 2), therefore with more fatigue the peak angular velocity and angular range were lower. These results suggest that a change of −2.78°/s in peak trunk angular velocity is associated with the experience of more physical fatigue, and similarly, a change of −1.15° in trunk angular range is associated with higher mental fatigue.

**Table 2:** Slope estimates and corresponding 95% confidence intervals for the associations that showed statistical significance

Type	Fatigue	Pct	Feature	Slope estimate [2.5%, 97.5%]	Relative magnitude	*p* value
sist	physical	95th	Peak trunk angular velocity (deg/s)	−2.78 [−4.78, −0.43]	−2%	0.003
	mental	95th	Trunk angular range (deg)	−1.15 [−2.06, 0.02]	−2%	0.015
stsi	physical	95th	PT duration (s)	−0.03 [−0.05, −0.01]	−1%	0.019
	physical	5th	Peak trunk angular velocity (deg/s)	1.27 [0.07, 2.54]	3%	0.027
	physical	5th	Peak vertical jerk (m/s^3^)	0.08 [0.00, 0.17]	2%	0.047
	physical	5th	Trunk angular range (deg)	0.88 [0.32, 1.67]	4%	0.0009

The relative magnitude represents the slope divided by the intercept.

*Pct* percentile, *sist* sit-to-stand, *stsi* stand-to-sit.

Similarly, the 95th percentile of the duration of stand-to-sit transitions was lower at higher physical fatigue, while the 5th percentile peak trunk angular velocity, peak vertical jerk and trunk angular range were higher at higher physical fatigue.

## Discussion

Fatigue has been recognised as a symptom that significantly affects HRQoL, particularly in people with NDD and IMID. One problem that has so far hampered the clinical assessment and monitoring of fatigue as well as the development of anti-fatigue therapeutics is the lack of objective measures of the symptom^50^. In this study the aim was therefore to quantify the association between IMU-derived kinematic features of sit-to-stand and stand-to-sit transitions and self-reported physical and mental fatigue. The hypothesis behind this was that physical fatigue, characterised by a lack of energy that hinders normal activities, is expected to impact muscle activity, especially in movements against gravity. In contrast, mental fatigue is likely to affect tasks requiring intersegmental coordination, like sitting down^51^. Different studies suggest that unsupervised daily mobility performance, derived from digital wearable technologies, is associated with fatigue^52–56^. These studies focused on quantitative (or ‘numerical’) measures of performance, such as the number of steps taken (which was associated with fatigue in PD^53^) or the time spent standing (which was associated with fatigue in RA^54^). To our best knowledge, the kinematic features presented in our study are the first movement-describing (or ‘qualitative’) performance measures for the objective assessment of fatigue.

The main findings of this study were that (i) various kinematic features of the transitions were associated with fatigue, (ii) only parameters of the upper and lower limits of performance, but not the median kinematic features (‘usual’ performance) showed significant associations with fatigue, (iii) more associations were found for stand-to-sit transitions than for sit-to-stand transitions, and (iv) more associations were found for physical fatigue than for mental fatigue.

Five kinematic features significantly associated with fatigue were related to physical fatigue and only one was related to mental fatigue. The predominance of the former could be explained by transitions being primarily a physical effort. Still, the observed negative association between the 95th percentile of sit-to-stand angular range and mental fatigue is interesting from a therapeutic point of view. This result suggests that increasing mental fatigue leads to less upper body flexion during standing for the movements with the greatest flexion. This could result in an increased risk of an unsuccessful transition (which can lead to falling back onto the chair or, in extreme cases, to a fall). It can be deduced that this mechanism should be investigated and specifically trained in people who fall during (effortful) sit-to-stand transitions. These people should also be specifically asked about the presence of mental fatigue. The other kinematic feature from the sit-to-stand transitions that was significantly associated with fatigue was the 95th percentile of peak angular velocity. This feature was negatively associated with physical fatigue. What this could mean is that when people are more physically fatigued, they have less physical capacity (or muscle strength) to stand up. Potentially comparable with our results, a recent study showed that the duration of sit-to-stand transitions was positively associated with self-reported fatigue in people with multiple sclerosis in a study using wearable sensors in daily life^50^.

Remarkably, twice as many stand-to-sit as sit-to-stand kinematic features were associated with fatigue. This may indicate that aspects of fatigue are better reflected by movements along, rather than against, the direction of gravity. The 95th percentile of the stand-to-sit duration showed a negative association with physical fatigue, which means that the longest transitions are shorter with increasing physical fatigue. In addition, the 5th percentiles of the peak angular velocity, peak jerk and trunk angular range were all positively associated with physical fatigue. Our interpretation of these findings is that when people are more physically fatigued, then they drop down in a more uncontrolled manner, resulting in a shorter duration and higher angular velocity and jerk of the sitting movement. It is of note that this was only true for the slowest stand-to-sit movements, but not for the fastest and normally paced movements, suggesting that the ‘drop down’ movements only occurred for those movements that were normally the slowest (or, in other words, the most controlled). Potentially clinically relevant information can also be derived from these observations. Firstly, people who report insecurity or even fear of falling / falls during the sit-to-stand movement should be examined for the presence of physical fatigue. Secondly, preventive and therapeutic opportunities may arise, for example, by working on the speed of the stand-to-sit movement.

According to our results, the 5th percentiles of the trunk angular range appears to be the most promising feature for progression and treatment response studies to record physical fatigue. This showed a 4% increase per item on the physical fatigue 7 items Likert scale, which suggests that if a person goes from low to high physical fatigue, that is from 0 to 6, then on average the 5th percentile of the trunk angular range increases by about 5°. This means that the movements with the least angular range show more angular range at higher physical fatigue than with less physical fatigue. Translating this to a real-world setting, it may mean that the movements of sitting down occur with a generally higher trunk angular range.

In a similar context, it has been shown that a decrease in transition duration of 0.3 s, a decrease in trunk tilt angle of 4° are associated with improved mobility in subjects with frailty after a rehabilitation programme^57^, and likewise an increase of 20° in trunk tilt angle and a decrease in peak angular velocity of 10°/s were found with the ability to rising from a chair^58^ as rated by the Movement Disorder Society-Sponsored Revision of the Unified. Parkinson’s Disease Rating Scale^59^.

One of the main findings of this study was that only the extreme values (5th and 95th percentiles), but not the median values, of the kinematic transition features showed statistically significant associations with fatigue. This finding was in line with previous information that extreme values for daily function assessment provide clinically relevant information regarding different domains of health. Most notably, the stride velocity 95th percentile, as measured from a shank-worn IMU^60^, has become the first wearable-derived digital clinical outcome assessment qualified by the EMA for use as a primary endpoint in trials for Duchenne muscular dystrophy^61,62^. Similarly, it was shown before that IMU-derived gait speed estimates in daily life provide complementary information in mobility assessment in PD^63,64^ and extreme values of gait characteristics were more informative of risk of falling in older adults^65^. In addition, in laboratory-based studies, frail elderly persons showed lower maximum and minimum accelerations than the physically active elderly persons in both sit-to-stand and stand-to-sit movements^66^, and similarly fallers showed slower vertical velocity, reduced vertical acceleration, lower vertical power and lower vertical jerk compared to non-fallers^42^.

We did not find significant associations between the median values of the kinematic transition features (which we defined as features of ‘usual’ performance^22^) and self-reported fatigue. At first glance, this seems surprising. The best way we can explain this may be that the usual performance of transitions is determined by the activities related to daily routine, such as groceries shopping, going to the toilet and going to work. These activities need to happen regardless of how fatigued the person actually feels. This ‘necessity of usual performance’ in everyday life may also explain why previous research also has not found a clear relationship between other aspects of usual performance, such as the amount of walking and fatigue^64^. The implication of these results for clinical practice and research is that, at least for the cohorts studied here, we cannot advocate the use of median kinematic features of sit-to-stand and stand-to-sit transitions for monitoring fatigue progression or treatment effects.

The study also suffers from some limitations, for example, physical and mental fatigue were assessed with a Likert scale ranging from 0 to 6. However, this score is subjective and the interpretation of the scale and what is meant by physical and mental fatigue may vary per subject, which makes it hard to compare responses on an inter-individual basis. In the literature, most studies prefer to use previously validated scales, such as the Functional Assessment Chronic Illness Therapy (Fatigue)^67,68^, that previously has been validated for RA^69,70^, SLE^71^, PSS^15,72^. It should also be noted that the definition of fatigue as such, and of mental and physical fatigue, is not uniform, so there is room for interpretation at this level too.

Furthermore, the current study explored cross-disease digital measures of fatigue^73^, however it is quite possible that there may be fluctuations within the different disease cohorts that could be explored in future work. Another limitation is that these findings are only relevant to the conditions studied here and to mobile people who do not use walking aids, for example. Finally, the statistical analysis is only explorative and the potential for overfitting and the risk of data overload are significant, as extraction of kinematic features from the IMU data generates an overwhelming number of metrics, making it difficult to identify which parameters are most relevant for assessing fatigue.

In conclusion, the aim of this study was to quantify the association between kinematic features of sit-to-stand and stand-to-sit transitions and self-reported physical and mental fatigue. To our best knowledge, this is the first study to show a relation between kinematic features of sit-to-stand and stand-to-sit transitions from a real-world dataset, and self-perceived physical and mental fatigue. The findings suggest that extreme values, i.e. 5th and 95ht percentiles of these kinematic features over predefined aggregation windows are associated with fatigue across different disease cohorts. Overall, these results highlight the potential of wearables-based assessment of non-motor conditions and deficits in the home environment of affected persons, to support the development of surrogate and even intermediate clinical endpoints that are objective and serve as a proxy for monitoring aspects of fatigue. This study may also motivate further studies in this area, including multi-sensor and multimodal approaches^2^ to assess this type of movement and also body function around this movement across different diseases^73^.

## Materials and methods

The IDEA-FAST project (https://idea-fast.eu/) aims to utilise multiple sensing modalities and technologies at home to identify digital health measures of fatigue in patients with Huntington’s disease (HD), PD, inflammatory bowel disease (IBD), primary Sjögren’s syndrome (PSS), rheumatoid arthritis (RA) and systemic lupus erythematosus (SLE). In this paper, the data were from a feasibility study^2,74,75^ during which participants reported about fatigue and digital devices were used for unobtrusive remote monitoring. The feasibility study assessed, amongst others, usability and acceptability of the different digital technologies, to be further tested in the subsequent clinical observational study (https://idea-fast.eu/deliverables/)^64,75^.

Ethical approval was first granted by the Ethical Committee of the Medical Faculty of Kiel University (D491/20) in June 2020 and then by the Research Ethics Committees of all other study sites: Newcastle-upon-Tyne Hospitals National Health Service Foundation Trust/Newcastle University in August 2020, Erasmus University Medical Centre in Rotterdam in November 2020 and George-Huntington-Institute in Münster in September 2020. The study was registered with the German Clinical Trial Registry (DRKS00021693) and was conducted according to the principles of the Declaration of Helsinki (version of 2013). All participants provided informed consent.

### Data collection

Study participants were consecutively recruited during routine clinical visits and through public outreach at information centres or support groups. Inclusion criteria^2^ included an age over 18 years, consent to participate in the study for up to 60 days and according to the study protocol, use of a smartphone in the past 3 months and ability to follow written and oral instructions in the local language, to walk, sit and stand independently and to socialise and communicate, as well as a score of over 15 points in the Montreal Cognitive Assessment (MoCA)^76^. Exclusion criteria were certain comorbidities like major sleep disorders, chronic fatigue syndrome, respiratory, cardiovascular or metabolic disorders or physical traumas with hospitalisation in the past 3 months, diagnosis of cancer in the past 3 years, major psychiatric disorders, suicidal attempt in the past 5 years or suicidal ideation in the past 6 months, substance or ethanol abuse or severe visual impairment.

The feasibility study was conducted at four sites, namely in Rotterdam (The Netherlands), Kiel (Germany), Münster (Germany) and Newcastle-upon-Tyne (United Kingdom), between July 2020 and December 2021. Participants were either healthy or diagnosed with HD, PD, IBD, PSS, RA or SLE according to the respective international guidelines^77–82^.

**Fig. 3:**
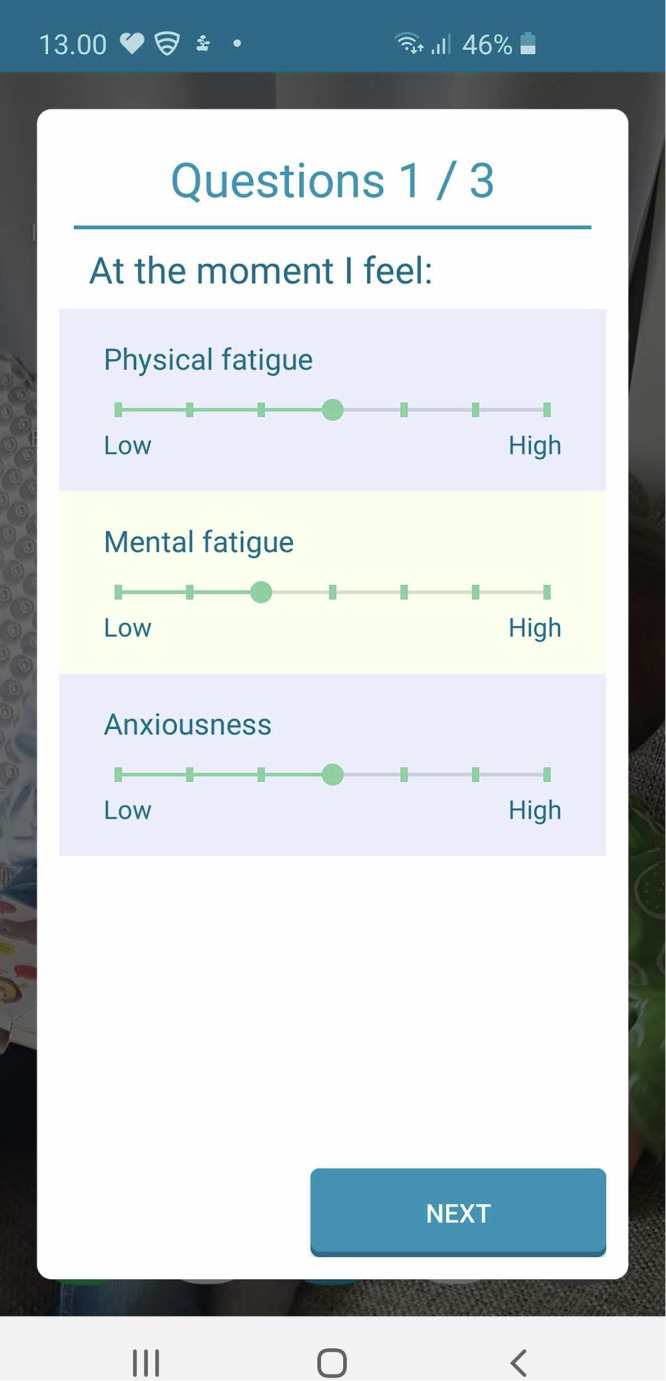
Stress Monitor App. The app interface for the four times a day patient-reported outcome on, among others, physical and mental fatigue.

PROs were collected using an Android smartphone application (VTT Stress Monitor App^83^,), that prompted users four times a day (i.e. at 9:00, 13:00, 17:00 and 21:00) to report, amongst others, their physical and mental fatigue^2^. The response could be submitted within 3 h of the prompted question, except in the evening as those responses were set due at 23:30. Median response time was 19 min. For the current study, the responses to ‘*At the moment I feel: Physical fatigue*’ and ‘*At the moment I feel: Mental fatigue*’ were considered, both of which were designed as a Likert item with seven options from low (zero) to high (six) fatigue^64^ (Fig. 3). The simultaneous collection of subjective and objective (surrogate) data of clinically relevant symptoms from the home environment over an extended period is the key novel aspect of this research. The definitions of fatigue, physical fatigue and mental fatigue were explained in detail to the study participants before the start of the study to ensure that each participant had a good understanding of these definitions.

Study participants were asked to wear an IMU device (Dynaport MoveMonitor, McRoberts BV, Den Haag, The Netherlands) on the lower back using an elastic strap for four consecutive weeks, and continue their daily life activities as usual. Participants put the device on themselves approximately at the level of the L5 vertebra. The IMU sampled tri-axial linear accelerations and angular velocities at roughly 100 Hz with the accelerometer range set to ±8 g and the gyroscope range set to ±1000°/s. No further calibration procedure was performed to reduce the burden on the study participants.

### Data processing

Data from the IMU were downloaded to a local computer and then further processed. For each file, the accelerations and angular velocities were resampled and interpolated to time-synchronise the recordings of the accelerometer and gyroscope at a constant sampling rate of 100 Hz. The data were then segmented per day, and the accelerations and angular velocities were used to identify postural transitions (i.e. sit-to-stand and stand-to-sit transitions) using an algorithm that has previously been validated in healthy adults, people with PD, people with multiple sclerosis, and people who have had a stroke^48^. In short, the algorithm first estimated the IMU’s orientation with respect to a global frame by fusing the accelerometer and gyroscope data^48,84^. The acceleration due to body movement is then obtained by subtracting gravity. Continuous wavelet transform was applied to the vertical acceleration to identify possible postural transitions^43,85^. The vertical displacement was estimated for each candidate postural transition, and a sigmoid function was fitted to the displacement curve. A candidate postural transition was considered a true sit-to-stand or stand-to-sit transition if both the R^2^ of the fitted model was above an empirically determined threshold and the elevation change was between a lower and upper bound^48^. From each postural transition, seven kinematic features were extracted to characterise the sit-to-stand and stand-to-sit transitions (Table 3).

**Table 3:** Overview of the kinematic features that were derived for each sit-to-stand and stand-to-sit transfer

Kinematic feature	Units	Description
PT duration	s	The time that it takes to complete the postural transition (PT).
Trunk angular range	degrees	The difference between the maximum and minimum tilt angle during a postural transition. The tilt angle was calculated by converting the quaternions to the Euler angles^48^.
Peak vertical velocity	m/s	The maximum vertical velocity during the postural transition.
Peak trunk angular velocity	degrees/s	The maximum angular velocity in the sagittal plane during flexion in a sit-to-stand transfer
Peak vertical acceleration	m/s^2^	The maximum vertical acceleration during the postural transition.
Peak vertical jerk	m/s^3^	The maximum vertical jerk (i.e. time-derivative of the vertical acceleration) during the postural transition.
Peak PT power	m^2^/s^3^	The maximum power normalised by the mass of the subject, i.e. the product of the vertical velocity and the acceleration during the postural transition.

*PT* postural transition.

The kinematic features were aggregated into statistical descriptors over 2-h windows. The windows were centred around the time at which a PRO was prompted. The sit-to-stand and stand-to-sit transitions for which any of the kinematic features’ value was greater than the value corresponding to the 99th percentile were discarded to ensure kinematic features’s values were in a reasonable range (i.e. extremely long transitions, of more than 4 s, were excluded)^48,49,86^. If there were at least three transitions, then the window was used for the analysis. The selected statistical descriptors were the median, 5th and 95th percentile^87^. The median values were believed to reflect the usual performance aspect of mobility, namely what a person ‘normally’ does in their usual environment, whereas the 5th and 95th percentiles reflect the upper and lower limits of this performance (which we call here ‘capacity-like’, as these extreme cases may reflect best the performance limits^63,65^.

### Statistical analysis

The association between the feature aggregates and the PROs were researched using mixed effects models^88,89^ with the kinematic feature modelled as dependent variable and the self-reported level of fatigue as the independent variable. For each kinematic feature, a random intercept and fixed slope model was calculated (i.e. the intercept could vary from participant to participant, but the slope was fixed across all participants). In total, this resulted in 42 regression models that we computed (two outcomes, seven kinematic features, three statistical aggregated descriptors).

In case the latter showed a slope estimate that was significantly different from zero (based on a significance level, *α*, set to 0.05), in addition, a random intercept, random slope model was calculated. Due to the presumed connection between two concepts of interest that span different components of the International Classification of Functioning, Disability and Health (ICF) model^90^ (fatigue as an aspect of body function and transitions as an aspect of mobility/activity), only small associations were expected. Note that Bonferroni correction and inclusion of potential confounders into the models was not used due to the explorative nature of the study’s aim.

## Data Availability

The data for this study are available from the corresponding author upon reasonable request.
